# Treatment outcomes of biologics and small molecules for chronic hand eczema: An evidence-based systematic review

**DOI:** 10.1016/j.jdin.2023.07.016

**Published:** 2023-08-15

**Authors:** Siddhartha Sood, Edgar Akuffo-Addo, Jorge R. Georgakopoulos, Asfandyar Mufti, Khalad Maliyar, Jensen Yeung

**Affiliations:** a Temerty Faculty of Medicine, University of Toronto, Toronto, Ontario, Canada; b Division of Dermatology, Department of Medicine, University of Toronto, Toronto, Ontario, Canada; c Department of Dermatology, Sunnybrook Health Sciences Centre, Toronto, Ontario, Canada; d Department of Dermatology, Women's College Hospital, Toronto, Ontario, Canada; e Probity Medical Research, Waterloo, Ontario, Canada

*To the Editor:*

Chronic hand eczema (CHE) is a debilitating heterogeneous condition characterized by pruritus secondary to hyperkeratosis, vesicles, or fissures.^1^ Although the initial treatment involves topical corticosteroids, currently there are no US FDA-approved targeted therapies for refractory disease.^1^ This systematic review examines evidence surrounding biologic and small-molecule treatment for CHE.

Following preferred reporting items for systematic reviews and meta-analyses guidelines, Embase and MEDLINE databases were searched using specific keywords (Supplementary Table I, available via Mendeley at https://data.mendeley.com/datasets/vtkgpr7c2m/1). Quality of evidence was assessed based on the Oxford center for evidence-based medicine 2011 levels of evidence. After independent screening by 2 reviewers, 33 articles (publication date: 2018-2023) reflecting 550 patients and 560 treatments with reported outcomes were included (Fig 1; Supplementary Table II, available via Mendeley at https://data.mendeley.com/datasets/vtkgpr7c2m/1). The mean age of the patients was 45 years (range, 12–79 years), with 218 males (39.6%), 321 females (58.4%), and 11 (2%) patients with unreported sex. The frequently reported clinical subtypes of CHE were as follows: atopic (22.4%, 123/550), irritant (21.3%, 117/550), hyperkeratotic (7.5%, 41/550), dyshidrotic (6.4%, 35/550), and allergic (3%, 17/550). CHE was refractory to nonbiologic or non–small molecule systemic or topical therapy in 94% (517/550) of patients.

**Fig 1:**
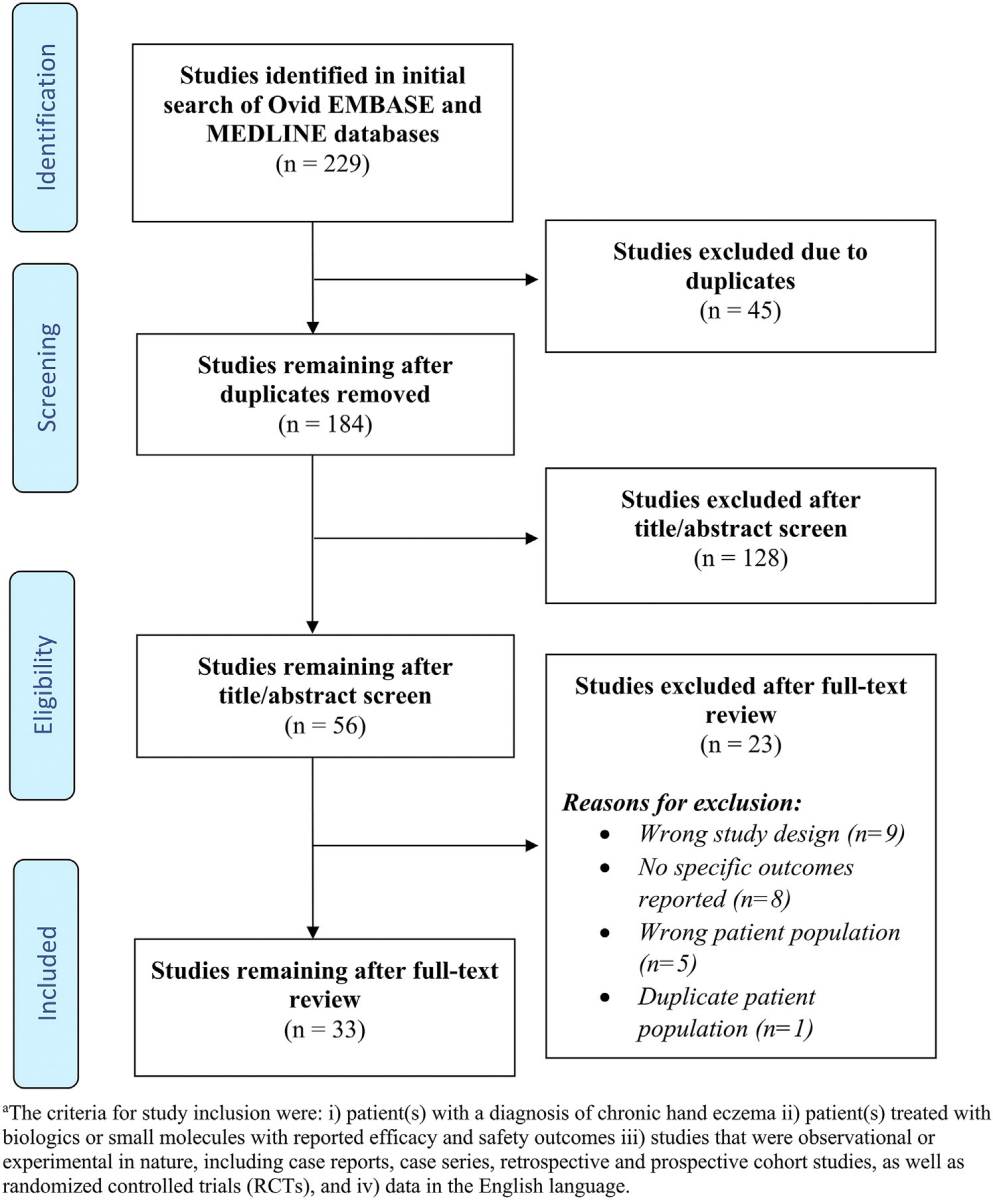
Flow diagram of literature screening using the preferred reporting items for systematic reviews and meta-analyses guidelines. (Figure adapted from http://prisma-statement.org.)

Mean treatment duration was 153.8 days in 524 of 560 patients. Concomitant medications were used in 23% (127/550) of patients, with topical corticosteroids (69.3%, 88/127) being the most frequent (Supplementary Table II). The most common biologic or small-molecule classes utilized were topical delgocitinib (40.1%, 228/560), dupilumab (34.5%, 193/560), gusacitinib (11.6%, 65/560), upadacitinib (6%, 34/560), topical crisaborole (3.2%, 18/560), baricitinib (2.1%, 12/560), and apremilast (0.8%, 5/560) (Supplementary Table III, available via Mendeley at https://data.mendeley.com/datasets/vtkgpr7c2m/1). Complete resolution was observed commonly with dupilumab (53.4%, 103/193), followed by crisaborole (38.9%, 7/18), gusacitinib (38.5%, 25/65), delgocitinib (35.5%, 81/228), upadacitinib (35%, 12/34), and baricitinib (33.3%, 4/12). Conversely, no resolution was documented often with apremilast (80%, 4/5). The studies used Hand Eczema Severity Index (HECSI) as a validated outcome measure wherein >41-point reduction from baseline may represent a meaningful improvement.^1^ A mean reduction of 89% (48.6 points), 87.9% (45 points), 77.2% (34.9 points), 68.9% (37.7 points), and 62.5% (38.6 points) in HECSI was observed with dupilumab, baricitinib, upadacitinib, delgocitinib, and gusacitinib, respectively, in 93, 2, 32, 228, and 65 documented cases (Supplementary Table III). Two (0.4%) patients on dupilumab experienced recurrence after the initial resolution of CHE. There were 258 (46.1%) treatment-emergent adverse events observed, commonly involving nasopharyngitis (13%, 73/560) and ocular surface disease (5.7%, 32/560). Of these, 15 of 560 (2.7%) discontinuations were required (Supplementary Table II).

Although the pathogenesis of CHE remains unclear, increased systemic Th1/Th2 activation along with upregulated interleukin-4 transcripts have been implicated in patients with several disease subtypes.^2^^,^^3^ These findings may explain the favorable outcomes observed with dupilumab, followed by pan-cytokine inhibition via Janus kinase targeting. Similarly, this may substantiate the difficulty in achieving control with conventional therapy.^1^ Evidence from clinical trials has demonstrated HECSI reductions of 88.1% and 72.1% with dupilumab and topical delgocitinib, respectively.^4^^,^^5^ These results are consistent with our review.

Study limitations include incomplete follow-up data and potential selection bias. Moreover, data heterogeneity prevented meta-analysis. Nonetheless, we highlight evidence that supports the use of biologics and small molecules such as dupilumab, delgocitinib, upadacitinib, and gusacitinib for CHE. Further larger-scale studies are warranted.

## Conflicts of interest

Dr Asfandyar Mufti has been a speaker for AbbVie and Janssen. Dr Jensen Yeung has been an advisor, consultant, speaker, and/or investigator for AbbVie, Allergan, Amgen, Astellas, Bausche, Baxalta, Boehringer Ingelheim, Celgene, Centocor, Coherus, Dermira, Eli Lilly, Forward, Fresnius Kabi, Galderma, Incyte, Janssen, LEO Pharma, Lilly, Medimmune, Merck, Novartis, Pfizer, Regeneron, Roche, Sanofi Genzyme, Sun Pharma, Takeda, UCB, and Xenon. The remaining authors Mr Sood, Mr Akuffo-Addo, Dr Georgakopoulos, and Dr Maliyar have no relevant disclosures.
